# Alberta Provincial Pediatric EnTeric Infection TEam (APPETITE): epidemiology, emerging organisms, and economics

**DOI:** 10.1186/s12887-015-0407-7

**Published:** 2015-07-31

**Authors:** Stephen B. Freedman, Bonita E. Lee, Marie Louie, Xiao-Li Pang, Samina Ali, Andy Chuck, Linda Chui, Gillian R. Currie, James Dickinson, Steven J. Drews, Mohamed Eltorki, Tim Graham, Xi Jiang, David W. Johnson, James Kellner, Martin Lavoie, Judy MacDonald, Shannon MacDonald, Lawrence W. Svenson, James Talbot, Phillip Tarr, Raymond Tellier, Otto G. Vanderkooi

**Affiliations:** Department of Pediatrics, Sections of Pediatric Emergency Medicine and Gastroenterology, Alberta Children’s Hospital, Alberta Children’s Hospital Research Institute, University of Calgary, Calgary, AB Canada; Department of Pediatrics, Stollery Children’s Hospital, University of Alberta, Edmonton, AB Canada; Provincial Laboratory for Public Health (ProvLab, Alberta Health Services), Departments of Microbiology, Immunology & Infectious Disease and Pathology & Laboratory Medicine, University of Calgary, Calgary, AB Canada; Department of Laboratory Medicine & Pathology, University of Alberta, Edmonton, AB Canada; Department of Pediatrics & Emergency Medicine, University of Alberta, Faculty of Medicine & Dentistry, Women and Children’s Health Research Institute, Stollery Children’s Hospital, Edmonton, AB Canada; Institute of Health Economics, Edmonton, AB Canada; University of Alberta, Edmonton, AB Canada; Department of Pediatrics, Alberta Children’s Hospital Research Institute, O’Brien Institute of Public Health, University of Calgary, Calgary, AB Canada; Department of Community Health Sciences, Alberta Children’s Hospital Research Institute, O’Brien Institute of Public Health, University of Calgary, Calgary, AB Canada; Departments of Family Medicine and Community Health Sciences, University of Calgary, Calgary, AB Canada; Department of Pediatrics, Section of Pediatric Emergency Medicine, Alberta Children’s Hospital, University of Calgary, Calgary, AB Canada; Department of Emergency Medicine, University of Alberta, Edmonton, AB Canada; Division of Infectious Diseases, Department of Pediatrics, University of Cincinnati College of Medicine, Cincinnati Children’s Hospital Medical Center, Cincinnati, USA; Departments of Pediatrics and Physiology and Pharmacology, Section of Pediatric Emergency Medicine, Alberta Children’s Hospital, Alberta Children’s Hospital Research Institute, University of Calgary, Calgary, AB Canada; Department of Pediatrics, Section of Infectious Diseases, Alberta Children’s Hospital, Alberta Children’s Hospital Research Institute, University of Calgary, Calgary, AB Canada; Alberta Health, University of Alberta, Edmonton, AB Canada; Alberta Health Services, Department of Community Health Sciences, University of Calgary, Calgary, AB Canada; Department of Pediatrics, University of Calgary, Edmonton, AB Canada; Faculty of Nursing, University of Alberta, Edmonton, AB Canada; Alberta Health Services, University of Calgary, Calgary, AB Canada; Division of Gastroenterology, Washington University, St. Louis, MO USA; Department of Microbiology, Immunology and Infectious Disease, University of Calgary, Calgary, AB Canada; Department of Pediatrics, Section of Infectious Diseases, Alberta Children’s Hospital Research Institute, University of Calgary, Calgary, AB Canada; Department of Pathology and Laboratory Medicine, Section of Microbiology, Alberta Children’s Hospital Research Institute, University of Calgary, Calgary, AB Canada; Department of Microbiology, Immunology & Infectious Diseases, Alberta Children’s Hospital Research Institute, University of Calgary, Calgary, AB Canada

**Keywords:** Vomiting, Diarrhea, Gastroenteritis, Norovirus, Rotavirus, Polymerase Chain reaction, Rectal swabs, Discrete choice experiments, Economic analysis, Vaccine

## Abstract

**Background:**

Each year in Canada there are 5 million episodes of acute gastroenteritis (AGE) with up to 70 % attributed to an unidentified pathogen. Moreover, 90 % of individuals with AGE do not seek care when ill, thus, burden of disease estimates are limited by under-diagnosing and under-reporting. Further, little is known about the pathogens causing AGE as the majority of episodes are attributed to an “unidentified” etiology. Our team has two main objectives: 1) to improve health through enhanced enteric pathogen identification; 2) to develop economic models incorporating pathogen burden and societal preferences to inform enteric vaccine decision making.

**Methods/Design:**

This project involves multiple stages: 1) Molecular microbiology experts will participate in a modified Delphi process designed to define criteria to aid in interpreting positive molecular enteric pathogen test results. 2) Clinical data and specimens will be collected from children aged 0–18 years, with vomiting and/or diarrhea who seek medical care in emergency departments, primary care clinics and from those who contact a provincial medical advice line but who do not seek care. Samples to be collected will include stool, rectal swabs (N = 2), and an oral swab. Specimens will be tested employing 1) stool culture; 2) in-house multiplex (N = 5) viral polymerase chain reaction (PCR) panel; and 3) multi-target (N = 15) PCR commercially available array. All participants will have follow-up data collected 14 days later to enable calculation of a Modified Vesikari Scale score and a Burden of Disease Index. Specimens will also be collected from asymptomatic children during their well child vaccination visits to a provincial public health clinic. Following the completion of the initial phases, discrete choice experiments will be conducted to enable a better understanding of societal preferences for diagnostic testing and vaccine policy. All of the results obtained will be integrated into economic models.

**Discussion:**

This study is collecting novel samples (e.g., oral swabs) from previously untested groups of children (e.g., those not seeking medical care) which are then undergoing extensive molecular testing to shed a new perspective on the epidemiology of AGE. The knowledge gained will provide the broadest understanding of the epidemiology of vomiting and diarrhea of children to date.

## Background

**Fig. 1:**
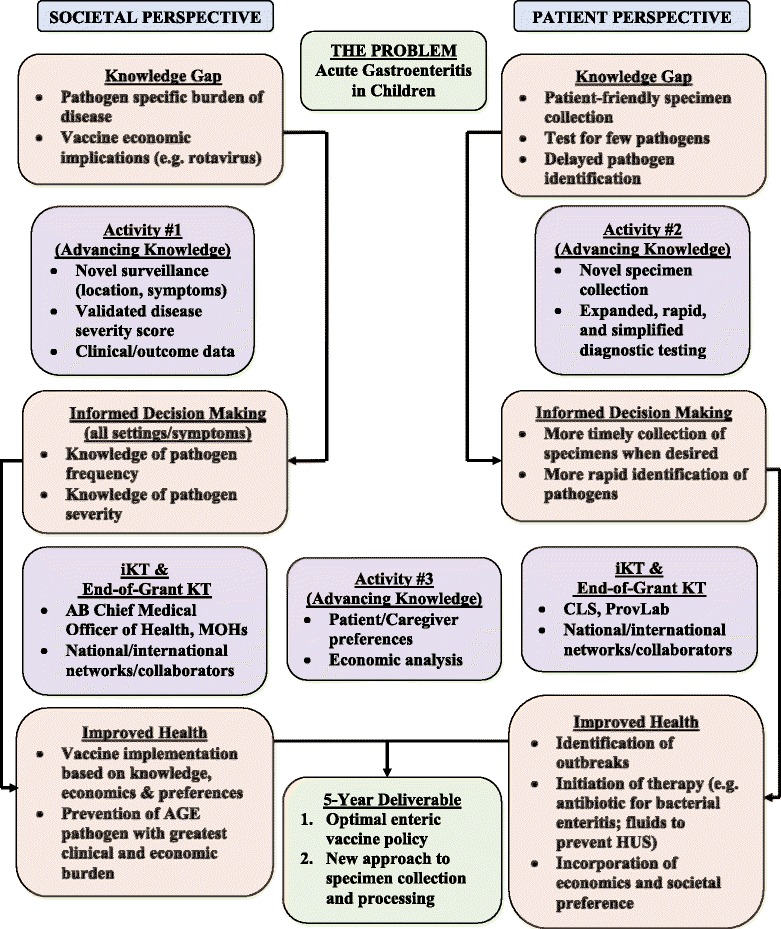
Pathway to Innovation

Current epidemiologic knowledge of acute gastroenteritis (AGE) pathogens is limited to subsets of children with select clinical presentations and by tests capable of identifying only a limited number of pathogens. APPETITE (The **A**lberta **P**rovincial **P**ediatric **E**n**T**eric **I**nfection **TE**am) has built a pathway to innovation (Fig. 1) that strives to develop a better understanding of the infectious causes of AGE by proposing a paradigm shift in specimen collection and testing. We will move from: **1)** stool collection (usually at home, if done at all, and returned to facility) to point of care swabs [oral/rectal – by health-care provider (HCP)]; **2)** multiple labour intensive tests (e.g., culture, microscopy, electron microscopy) with poor sensitivity to a single device, which employs polymerase chain reaction (PCR) technology to target multiple pathogens with high sensitivity and has the potential to be integrated into point of care testing technology [1–3].

Our proposal includes three activities that are chronologically inter-woven and interconnected to maximize the conduct of integrated knowledge translation (iKT). The activities are: **Activity 1** - **burden of disease assessment** through broad population surveillance with expanded data collection; **Activity 2 - enhanced pathogen identification** through improved specimen collection (oral and rectal swabs) and molecular (PCR) diagnostic technology; and **Activity 3 – preference elicitation and economic modeling** which will be planned, and interpreted alongside our decision-makers. Activity #3 will be conducted in conjunction with the data and knowledge gained from Activities #1 and 2. Multi-directional knowledge exchange will be used to address the following key questions identified by our end-users: Theme 1) Do we add an enteric vaccine to Alberta’s vaccination schedule, and when (now for rotavirus; later for norovirus)? If so, how do we optimize uptake?; Theme 2) Do we change policy and employ swabs in Alberta’s laboratories that process specimens? Do we integrate multi-analyte, non-culture PCR technology into all or some of our laboratories? What would the cost be of such an approach?

APPETITE’s research plan was designed through a series of in-person meetings, teleconferences, and an in-person all day gathering of investigators in Calgary (October 28, 2013). APPETITE’s agenda addresses a high-impact, high-needs problem while balancing the needs of our end-user partners. The agenda directly tackles key health policy issues - child health, infectious disease, health promotion, vulnerable populations - identified by Alberta’s Health Research and Innovation Strategy [4], and Alberta’s Maternal, Newborn, Child & Youth Health Emerging Clinical Network’s [5] research priorities (i.e., rotavirus immunization).

Suggestions from the Alberta Children’s Hospital (ACH) Family Advisory Committee (November 7, 2013), and a HCP survey (November 2013) were integrated into our research plan. Preliminary analysis (n = 90) provided the following insights: 1) 53 % believe caregivers would be interested in adding an enteric vaccine; 2) 81 % believe an additional requirement should include cost-effectiveness; 3) only 9 % believe that current methods of stool sample collection are easy and convenient; and 4) 82 % believe that specimen submission would be improved if rectal swab samples were employed.

### Knowledge gaps

#### We test for too few pathogens in too few patients and thus do not understand the true impact of AGE

1. Unknown Epidemiology of Disease (Fig. 2):
  Although 90 % of affected individuals do not seek medical care, they account for over 70 % of the total cost of illness (due to missed employment) [6]. Of those who do seek care, only 6 % submit stool samples [7]. Even then, only diarrheal specimens are tested and most often for bacteria and parasites (Table 1), and such agents cause only 10-15 % of disease. Over 60 % of pediatric stool cultures in Calgary are from hospitalized children who represent <1 % of the pediatric population with AGE [8, 9]. In a survey of 1000 randomly identified patients at 31 Canadian emergency departments (ED), vomiting was the most common reason children were brought for care [TRanslating Emergency Knowledge for Kids (TREKK); http://trekk.ca/]. [10]. However, because of technological limitations children with isolated vomiting (i.e., without diarrhea), rarely have samples tested to identify an infectious etiology. Hence diarrheagenic pathogens are over-represented among causes of enteric events. Our lack of true pathogen-specific burden of disease knowledge impairs our ability to deliver evidence-based prevention strategies.

**Table 1:** Results from routine pediatric laboratory testing (Calgary Laboratory Services); 01/01/2012 – 31/12/2012

Calgary Laboratory Services	# Performed	% Positive
Viral testing (Rotavirus only)	1541	10 %
Bacterial testing	3472	6 %
Parasite testing	3230	9 %
*Clostridium difficile* testing	2071	12 %
Totals	10,314	8.7 %

Viruses, responsible for 70–90 % of AGE, were only identified in 10 % of samples. The most common tests – bacterial culture and parasite microscopy, had the lowest rates of positivity

**Fig. 2:**
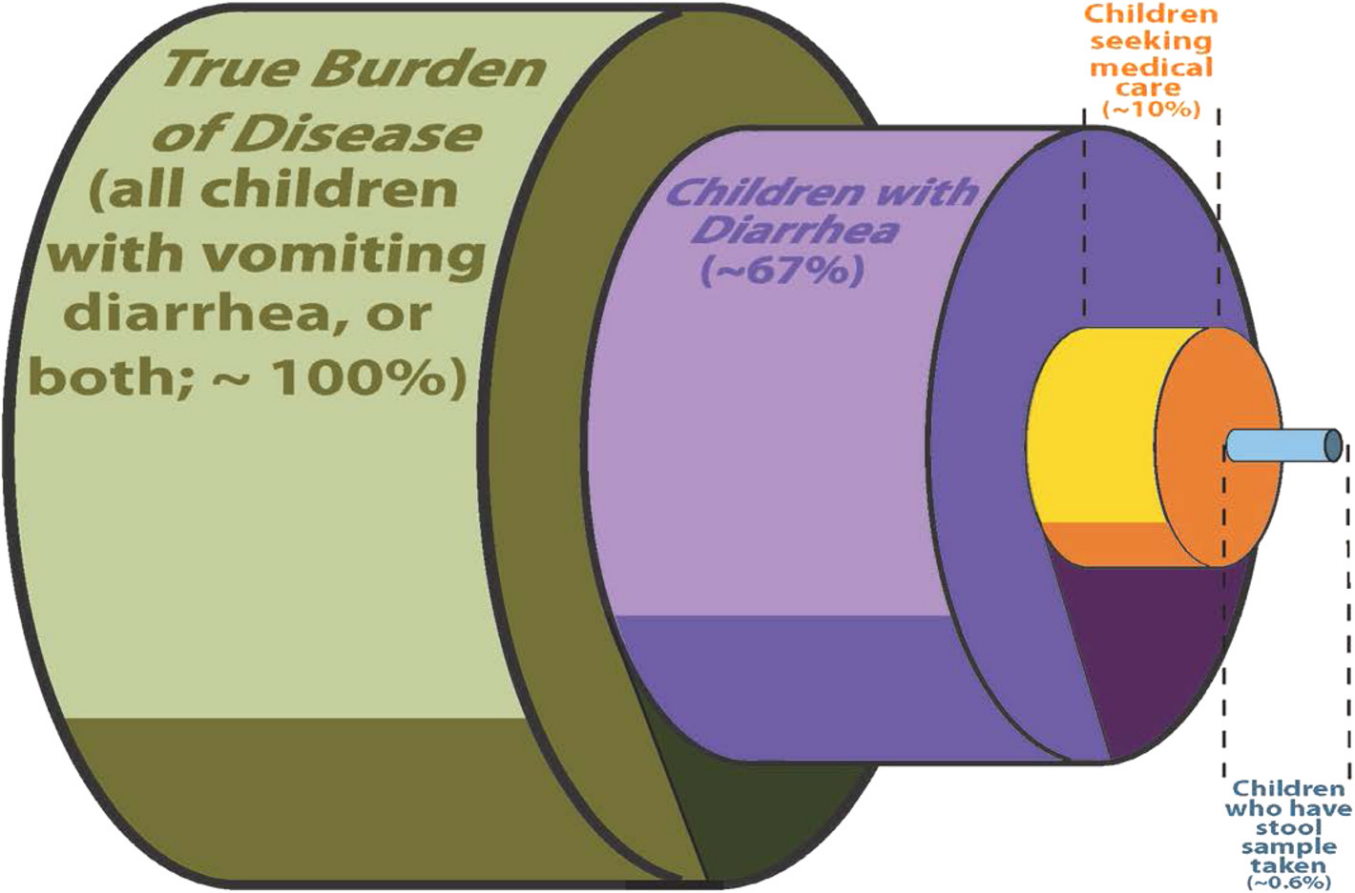
Overall societal burden of disease in relation to those who undergo testing. The image portrays the total burden of acute gastroenteritis (green), in relation to the smaller subset that have diarrhea (only group eligible for testing; purple). This is followed by the much smaller proportion who actually seek medical care (yellow) and lastly by the tiny proportion who actually undergo testing (note, even when tested, standard testing is limited by the ability to identify the organisms causing 20 – 30 % of disease)
2. Pathogen Identification
  Enhanced enteric disease diagnostics are needed as 1) there are significant public health implications; 2) the potential severity of disease is significant (2–3 % of those affected develop persistent health problems) [11]; and 3) timely treatment may be crucial. Examples of the importance of pathogen identification include: 1) Verotoxin-producing *E. coli* (synonymous with Shiga-toxin producing *E. coli*) [12], although uncommon, can cause hemolytic uremic syndrome, the most common cause of acute renal failure in children [13, 14]. The lifetime cost/case is >2.8 million Euro [15]. Current testing algorithms entail sending patients home to collect and return stool specimens. If returned, often 1 – 2 days later, such specimens are processed employing culture techniques, which further delay pathogen identification [16]. Point of care collection (i.e., rectal swab) followed by rapid molecular identification (e.g., PCR) would enable the early provision of therapies to reduce the severity of renal failure [17–19]; 2) The identification of norovirus, which causes 800 deaths annually in the U.S. [20], may lead to passive immunotherapy administration to high-risk individuals [21]; 3) From a public health perspective, a rapid assessment tool will assist in separating epidemic episodes of mild AGE from public health emergencies [22].
3. Patient Preferences and Cost Effectiveness
  Little is known about preferences of patients and caregivers in relation to enteric vaccines. Economics also play a role in decision-making processes because optimizing the distribution of limited health-care resources is mandatory [23]. In accordance with recommendation in the 2013 report from the Public Health Agency of Canada [11], we conducted focus group meetings with the ACH Family Advisory Committee. The following themes emerged from these discussions: 1) need to focus on introducing “critical” vaccines; 2) a vaccine now (i.e., rotavirus) may decrease future vaccine uptake (i.e., norovirus); and 3) importance of comparing public health impact of rotavirus vaccine to other (non-AGE) potential interventions that could be implemented. However, since studies to date have not included children with isolated vomiting, which is commonly seen with norovirus [24, 25] and those not seeking care, the economic models employed to guide vaccine policy, and outline the opportunity costs of such policies, have employed data with significant limitations [26–28].

## Methods/Design

### Overview

This phase of our team’s study focuses on developing an adaptation-friendly reproducible model of infectious disease epidemiology assessment. It is designed to capture a comprehensive clinical picture of AGE (i.e., those with isolated vomiting, isolated diarrhea, and combined) in diverse settings (e.g., home, primary care, ED). Evaluating etiology in an unbiased manner has been identified as priority by key decision-makers to 1) identify the most common pathogens (viral, bacterial, parasitic); 2) associate each pathogen with illness severity (all settings); and 3) enable the construction of economic models to inform health-care investment decisions.

### The health challenge

**Fig. 3:**
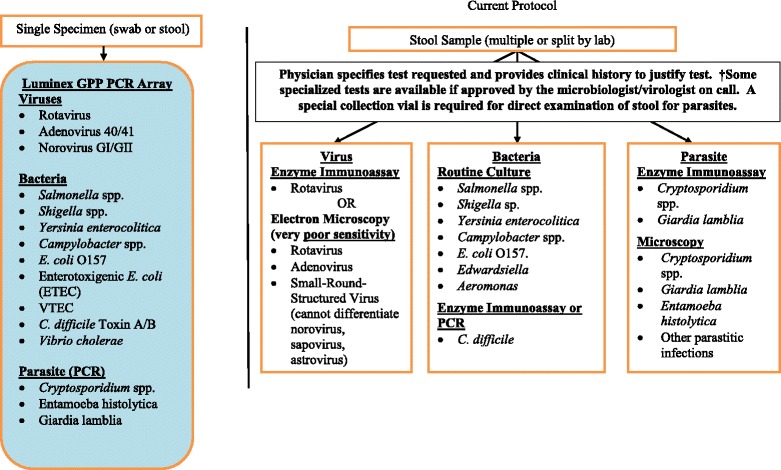
Current testing compared with multi-analyte array. Clinical testing (right) is limited to a narrow group of pathogens (unless multiple tests are ordered). The Luminex GPP multi-analyte array we propose to use (left) employs a single stool or swab (rectal/oral) specimen to perform a comprehensive test panel. The results will delineate the epidemiology of AGE in Alberta (Activity #2; left). Note - VTEC (non O157), ETEC, and the majority of viral pathogens are not identified under current testing algorithm (right). Note - *Edwardsiella* is only routinely tested for in select laboratories in the province

We do not know which pathogen(s) are most responsible for AGE in terms of frequency or severity because the full community-based spectrum and extent of AGE (which is 8 times more frequent than physician visits) [29] has not been performed in recent decades. The frequency of AGE and its impact in the community is reflected in Health Link Alberta (provincial telephone help-line) data: 39 % of all pediatric calls are because of AGE and 67 % result in a recommendation for care at home [courtesy Alberta Health (AH)]. Although viral infections cause 75–90 % of infectious gastroenteritis, current testing approaches identify a pathogen in only 10 % of specimens (Fig. 3) because testing focuses on rotavirus which has been viewed as the most important pathogen [30]. Consequently rotavirus has been the target of Canadian surveillance efforts [31] which collect data on hospitalized children and select ED populations. In light of this knowledge gap, the Office of the Chief Medical Officer of Health of Alberta has added rotavirus to the notifiable diseases list effective January 2014. However, standard clinical testing will remain restricted to individuals with diarrhea presenting for ED care and those requiring hospitalization. In the current state, the true burden of disease (e.g., isolated vomiting; never tested) will remain unknown as will the prevalence of other pathogens, especially norovirus [32].

Alberta is an outstanding setting in which to study the pathogen-specific burden of disease because 1) rotavirus vaccine has not been introduced in this province so the natural spectrum of illness exists; 2) vaccines are administered at public health clinics thereby providing a wealth of population and patient specific vaccine data; and 3) our team will have access to clinical outcome and exposure data related to notifiable enteric pathogens. The need to study AGE epidemiology where rotavirus remains prevalent is important. In U.S. where rotavirus vaccination is common, norovirus is the most frequent cause of diarrheal disease in children [33–38]. However, the relative burden of disease (rotavirus vs. norovirus) in the absence of rotavirus vaccine is unknown.

### Project #1: case definitions

Case definitions for illnesses likely to be caused by each candidate pathogen will be defined *a priori.* This work will employ a modified Delphi technique consisting of anonymous questionnaires and a face-to-face expert panel meeting. This is required as not all positive tests represent true “disease” (e.g., *C. difficile* in children <1 year rarely causes disease) [39].

#### Indicator identification and development

For the clinical features selected by the advisory panel, a review of the literature will be conducted to identify 1) existing case definitions 2) practice guidelines, and high quality systematic reviews that could be used to inform the creation of case definitions and 3) infections for which no guidelines or evidence exists.

#### Existing case definitions

Existing case definitions for the selected pathogens will be identified through a search of the literature on enteric infections in children. Search terms will include those corresponding to clinical features and case definitions combined with terms describing each of the enteric pathogens. Examples of search terms will include: case definition, clinical feature, specific pathogen and infection/carrier. An initial list of existing case definitions and references will be created. Case definitions will be presented by pathogen, infection status (carrier/”infection”), and diagnostic test modality.

Existing case definitions for the selected conditions will also be identified by searching websites that focus on enteric infectious diseases and specific pathogens. Examples of websites include the Infectious Disease Society of America.

#### Advisory panel

An advisory panel consisting of approximately 20 decision makers and stakeholders will be created to select variables to be included in our case definitions. The Delphi technique, a structured interactive method involving repetitive administration of anonymous questionnaires, will be used [40]. The advisory panel will consist of pediatricians, pediatric emergency medicine physicians, infectious disease experts, medical microbiologists, and experts in virology and laboratory medicine. Participants will be identified by APPETITE’s International Scientific Advisory Committee and Strategic Advisory Committee and will be recognized experts in this field and will be representative of the perspectives held in Canada, the USA and Europe. Representatives from stakeholder organizations will be contacted and asked to provide the names of individuals with an interest in pediatrics, infectious disease and enteric infections. The panel will then be chosen from the master list of experts by the research team. Criteria for selection will include: interest or expertise in enteric infections in the pediatric population, experience in infectious diseases, geographic representation across Canada, practice diversity, and balance between individuals with clinical expertise and those with laboratory expertise. Selected panel members will receive a written invitation to participate in this project that will outline the goals of the project, their responsibilities and the timeline. Written invitations will be followed by a telephone conversation with one of the study investigators.

A description of the goals of the research project, as well as a summary of the literature review including the full list of existing and newly developed evidence-based, case definitions will be sent. The case definitions will be organized by pathogen, specimen type (rectal swab, oral swab, bulk stool), and diagnostic test employed.

The advisory panel will be sent the candidate list of variables to be considered. They will then be asked to suggest other clinical features that might assist in discriminating “infection” from the “carrier” state. A complete list of all candidate clinical features will then be assembled. A prioritized list of clinical features will then be generated through a multistage survey process.

In the first stage of the survey panelists will be asked to rate each clinical feature on three different criteria: 1) Importance/Impact – to what degree is this clinical feature important when discriminating infection from carrier state. 2) Prevalence – how frequently is this clinical feature present in those usually determined to have “disease”. 3) Validity – how solid is the scientific or professional consensus to support a link between the clinical feature and the presence of “disease”. Panelists will be asked to rate each clinical feature on all three criteria on a scale from 1 (strongly disagree) to 9 (strongly agree).

Any clinical feature rated 1, 2 or 3 for all of the 3 criteria by all panelists will be removed from further consideration. Any clinical feature rated 7, 8 or 9 by all panelists for all 3 criteria will be retained for further case definition development and removed from the next survey round.

The remaining clinical features will be included in a new survey for the second stage of the Delphi Process. The second survey will be individualized for each panel member and for each clinical feature and will include the member’s prior rating, the overall panel median rating and the range of ratings assigned on each of the clinical features. The panelists will then be asked to re-rate each clinical feature on the same three criteria. Each panelist’s ratings for the three criteria will be added. Conditions with mean scores ≥7 by 2/3 of panelists will included in the final list of clinical features used to define “disease”.

The advisory panel will then have a face-to-face meeting. At the meeting, the panelists will review anonymized ratings of all the clinical features. Panelists will be asked to refine highly scored clinical features and to discuss low scoring and indeterminate clinical features. Panelists will also be given the opportunity to suggest clinical features for pathogens with no existing case definitions and to identify significant gaps where more knowledge is needed. At the end of the face-to-face meeting panelists will be asked to independently re-rate each clinical feature on the same four criteria. Clinical features with mean scores ≥7 across all four criteria by 2/3 of panelists will be included in the final list of indicators.

### Project #2: specimen collection/testing

We will collect specimens (oral and rectal swabs plus stool) from children spanning the spectrum of severity and clinical presentations (i.e., isolated diarrhea, diarrhea and vomiting, isolated vomiting). A multi-year evaluation will allow us to capture the seasonal variation of clinical presentation and intensity of pathogen activity [41, 42].

#### Objectives

The project will achieve the following objectives:

1. Determine if swab specimens are adequate (or even preferred) alternatives to stool sample collection and testing, stratified by clinical presentation, for viral, bacterial, parasitic and *C. difficile.*
2. Determine the test characteristics of the multi-analyte assay employing all three sampling methods, in comparison with reference standards (i.e., stool culture), and work with end-users [Calgary Laboratory Services (CLS), Provincial Laboratory for Public Health (ProvLab)] to clarify its role in the Alberta context.
3. Determine the genotype profiles of bacteria and viruses in relation to vaccines and epidemic strains.

Note – oral swabs will only be analyzed in the context of viral pathogens (no expectation to identify bacteria, parasites).

#### Outcome measures

*Primary outcome:* The proportion of all cases caused by the most common pathogen (likely rotavirus or norovirus).

*Secondary outcome:* The ‘*burden of disease index*’ which is created by multiplying 1) frequency of disease and 2) mean Modified Vesikari Scale score to provide the burden in relation to setting and resource use.

#### Study population

Inclusion Criteria: Eligible children will:

1. Be aged 0–17.99 years of age
2. Have AGE defined by the presence of a minimum of three episodes of vomiting OR diarrhea in the preceding 24 h and < 7 days of symptoms [43] (*Medical Care*)

Exclusion Criteria: Children with any of the following will not be eligible:

1. Previously enrolled within the last 14 days (*Medical Care*)
2. Previously enrolled in the study (*Self-Care*)
3. Unavailable for follow-up
4. Chief complaint likely related to acute infection (i.e., presence of any of the following: vomiting, diarrhea, fever, running nose, and/or cough) (*Self-Care*)
5. History of depression, bipolar disorder, schizophrenia, or other significant underlying psychiatric illness
6. History of low white blood cells (neutrophils < 1000)
7. The child needing emergent care from the clinical team

Settings

Participants will be indentified from two cohorts:

1. Medical Care (N = 2250): To reflect a broad range of severity, children will be recruited from EDs including Alberta’s two pediatric EDs (Calgary and Edmonton). Both are members of Pediatric Emergency Research Canada (PERC) which has endorsed this study. Specimens (stool, oral and rectal swabs) will be obtained from children during the ED visit (if consent and assent are obtained as appropriate-Appendix 5). Home stool collection will be performed for those unable to provide a sample while in the ED and will be achieved by providing families with collection kits, instructions for specimen handling and preparation and a number to call to arrange for specimen retrieval and transport for processing (Appendix 5) [44].
2. Self-Care (N = 2250): This group will illuminate the burden of disease amongst children whose families do not physically seek evaluation. This cohort represents the greatest burden of disease, particularly in economic terms (i.e., caregiver lost productivity). We will recruit (i.e., pre-enrol) these participants while asymptomatic to enable them to participate when they experience an episode of AGE (defined as ≥3 episodes of vomiting or diarrhea in a 24 h period) [43]. Based on available data, 90 % of such events will not result in medical care [45] – such visits will define our *self-care* cohort. Should medical care be sought (10 % of this group), they will be re-assigned to the *medical care* cohort. At the index visit, children will have rectal and oral swabs performed to enable the generation of baseline data to understand the carrier rate in the general population. This opportunity will also serve as a teaching moment such that caregivers will understand how to collect specimens (oral and rectal swabs, and stool) at home once their child becomes ill. Prior to leaving the ED, they will be provided with collection kits, instructions for specimen handling and a number to call to arrange for specimen retrieval and transport for processing (to optimize return rate) [44].

In addition children will be recruited through HealthLink Alberta. This is a telephone resource, which is accessible to all Albertans, that provides guidance on the management of common medical conditions. For true emergencies, callers are told to call 911. For very minor conditions they are given guidance and instructed to manage the client at home. For those with symptoms of unclear importance they are often instructed to seek medical care. HealthLink AB has agreed to provide, with caregiver permission, the contact information related to phone calls of children aged < 18 years with AGE who are instructed to be cared for at home. The APPETITE team member will then follow-up with the caregiver/child to seek consent/assent for participation. This can be done either 1) verbally by phone; or 2) electronically with the consent/assent forms downloaded from the study website (www.gotgastro.ca) and then returned to our team electronically. Once consent is obtained the study sample collection packets and instructions will be emailed to the client/caregiver and they will be asked to complete the data collection forms.

The medical care and self-care cohorts will have clinical data collected:

1. In person or electronically/telephone on the day the specimens are provided/collected (Appendix 8).
2. 14 days after initial specimen collection (Appendix 9). This approach will enable us to collect outcome data to quantify illness severity employing the Modified Vesikari Scale score. This validated score [46, 47] is based on symptoms and therapy provided and is designed for use in AGE of unspecified etiology. Additional data collected will include risk factors for infection and severe disease, expenditures and quality of life.

#### Experimental maneuvers – medical care cohort

Patients will be assessed and treated by ED staff without any regard to this study. Stool specimen testing will continue as per standard of care at all participating sites. Once the inclusion criteria are confirmed and parental consent is obtained (and child assent when appropriate) an enrollment ID number will be assigned. Research ethics board approval was obtained for recruitment and specimen collection from both the Conjoint Health Research Ethics Board of the University of Calgary, and the Health Research Ethics Board of the University of Alberta.

#### Index interview

Parents will be asked to participate in a standardized 5 min survey administered by study personnel. The survey will collect descriptive data regarding gastroenteritis symptoms, childcare arrangements, parental work data, medical treatment sought during the child’s illness, and personal medical history. Those enrolled in an ED will directly enter the information into an iPAD device.

#### Specimen collection

We will collect four specimens (2 rectal swabs, 1 oral swab, and a stool sample) from each enrolled child. Approximately 750 children will be enrolled annually from our medical care cohort. Oral and rectal swabs will be performed prior to departure from the point of care using a flocked rectal swab (FLOQSwab, Copan Italia, Brescia, Italy). Stool will also be collected prior to departure. If a specimen is not provided prior to departure, we will provide caregivers with specimen collection containers and contact information for a courier service that will retrieve the sample. After cultures are performed, all swabs and stool specimens will be frozen at −70 °C for future testing which will be performed in batches.

#### Follow-up

At the index visit, caregivers will be asked their preferred method of communication – electronic (i.e., email survey) versus telephone. 14 days after enrollment, a standardized script or survey/data collection form will be employed to conduct follow-up. This would be performed using a secure RedCAP data system to minimize time required and transcription errors. If phone is opted for, the caller will enquire about ongoing symptoms, medical evaluations, treatments, child care and work absenteeism, and side effects. Detailed questioning will follow positive responses. The survey will employ advanced logic to enhance ease of use. Electronic surveys will be e-mailed daily (maximum of 3 times) until a survey is completed. If the caregiver does not complete the electronic survey after 3 days, telephone follow-up will be performed. Protocols will be developed to deal with caregiver questions in accordance with institutional requirements.

All standardized surveys employed in this study consist of a series of single-and multiple-select closed-ended items allowing for the selection of a residual “other” category. In cases in which the respondent feels that his or her response does not correspond to one of those already listed (ie. he/she selects “other”), the respondent will be asked to elaborate on his or her answer in full text.

In order to optimize follow-up the following data is collected at the initial point of contact: home, cellular, work and alternate telephone numbers; e-mail and home address. If phone follow-up is performed, we will call all numbers provided at least once daily over 3 days (maximum 5 attempts).

#### Specimen testing

In Year #1, all stool and rectal swab specimens [48, 49] will be analyzed employing the following:

1. RT-PCR 5- target ‘in-house’ virus panel (adenovirus, astrovirus, norovirus, rotavirus, sapovirus) [29];
2. multi-analyte assay [Luminex xTAG® Gastrointestinal Pathogen Panel (GPP)]
3. standard bacterial culture

The Luminex GPP is an FDA and Health Canada approved, qualitative bead-based multiplexed molecular diagnostic test that simultaneously identifies AGE causing viral, bacterial and parasitic pathogens. It has excellent test characteristics when stool^97,125^ and rectal swabs^100,104^ are employed - sensitivity >94 %, specificity >98 % [50, 51]. Moreover, 65 % of pathogens identified by the Luminex GPP remain unidentified when current laboratory procedures are followed (i.e., physician must suspect and order the appropriate pathogen specific test) [50]. We will explore the use of the Luminex GPP to identify viral targets from *oral swabs* which will undergo confirmatory analysis via our ‘in-house’, RT-PCR, 5-target viral panel. Employing Plan-Do-Study-Act cycles we will modify our protocols (collection methods, swab types, transport media, nucleic acid extraction) based on results. Pathogen detection accuracy will be compared, as appropriate, between the varying specimen collection methods. The ‘best’ (accurate and convenient) specimen type for testing will be identified. Once optimal specimen collection type and analysis methods have been determined, subsequent testing will be streamlined.

Because monitoring for genetic and antigenic changes in pathogens is crucial for viruses [52], specimens testing positive for rotavirus will be analyzed for G typing (VP7) and P typing (VP4) using nested RT-PCR [53]. Those positive for norovirus will be typed by DNA sequencing [52, 54]. Typing of bacterial targets will be performed, as appropriate, using standard methodologies [55] to evaluate the ability of our surveillance and comprehensive diagnostic approach to discover outbreaks not identified by traditional approaches.

#### Experimental maneuvers – self care cohort

Screening, consent and the index interview will occur as per the medical cohort.

#### Specimen collection

We will collect three specimens (2 rectal swabs, 1 oral swab) from each enrolled child during the index visit. Approximately 750 children will be enrolled annually from our self- care cohort. Oral and rectal swabs will be performed prior to departure from the point of care using a flocked rectal swab (FLOQSwab, Copan Italia, Brescia, Italy). After a culture is performed, all swabs will be frozen at −70 °C for future testing which will be performed in batches.

#### Repeat specimens

At the index visit, caregivers will be provided with specimen collection containers, and instructions on sampling to be repeated when their child does become sick. They will be instructed to contact a research team member should their child have ≥ 3 diarrheal or vomiting episodes in a 24 h period. This contact will initiate a call to a courier service to conduct specimen retrieval along with the e-mailing (or telephone completion) of the baseline illness survey. If the caregiver does not contact a team member, follow-up reminders will occur three and/or six months following the index visit.

Follow-up and specimen testing will occur as per the medical care cohort.

### Sample size

We anticipate that rotavirus and norovirus will each be present in ~20 % of cases. All other viruses combined will be identified in ~20 % of cases; bacteria may be present in 10 %. Thus, a pathogen will be identified in 60 – 70 % of participants. Each cohort (*medical care & self-care*) will require 2250 (4500 in total) participants assuming a point estimate of 20 % for the most frequent pathogen (closest to point of most uncertainty to ensure accurate estimates for less frequent ones) and a desire for the 95 % confidence interval to be ≤ ±2 %. The prevalence of disease attributable to each pathogen will be stratified by zone, potential risk groups (e.g., aboriginal, co-morbidity, age, socioeconomic status) [43], exposures, season, enrollment location, symptoms, severity, site of recruitment, and antibiotic use. 4500 cases (and ~3150 containing at least one of the pathogens tested in this study) will allow for a minimum of 10 cases of AGE for each stratification covariate and thus extensive regression modeling. Since the primary outcome is based on point-of-care rectal swab performance, compliance is a potential concern for the self-care group. Recruitment for the later will continue until specimens are received from the required number of participants (N = 2250). Duplicate samples <14 days from the same individual will be removed to provide case-based data

Clinical features and outcomes will be correlated with microbiologic findings. Age and sex-adjusted pathogen specific prevalence and severity data will be calculated and compared for all targets. Seasonality and geographic distribution of each pathogen over time will be analyzed.

### Integrated knowledge translation plan

Results will be analyzed bi-weekly, and the formats and fields will be tailored to end-user needs. Recruitment strategies will be modified in real-time based on success (e.g., add/drop clinics, study promotion). Education targeting participating families (e.g., information sheet, hydration, hand hygiene, transmission) will be central to our research-practice loop and will be refined based on accrued data. Through our knowledge/end-user collaborators, and utilizing the communication and reporting chains present within their respective organizations (primary care network leaders, pediatric ED end-users, informatics, public health officers) we will share and exchange findings to integrate results immediately to assist with unrecognized outbreaks, pathogen clustering, severe disease and complications. We will maintain bi-annual dialogue with our partner parent group to modify our data collection and aims. There will be ongoing discussion of our results at network meetings [PERC, TREKK, National Advisory Committee on Immunization (NACI), medical officers of health, Alberta Health Services (AHS), AH] and immediate improvements in health-care delivery could result through early implementation of novel sampling and testing methods or by linking of ED physician and nurse staffing to weekly APPETITE data.

## Discussion

### Governance

APPETITE has identified the need for a well designed governance structure and research management mechanism. The governance model is designed to enhance cross-sectoral collaborations and partnerships between academia, industry and the applied healthcare setting. The entities within the model create a framework whereby APPETITE leaders are challenged with rigorous internal checks and balances on research accountability, quality, and productivity. The governance structure is comprised of the following groups:

#### Strategic Advisory Committee (SAC)

Advises the APPETITE Executive Committee on the overarching plans and goals at a high level (e.g., major projects, activities, future funding, business model). The SAC is comprised of representation from APPETITE’s provincial [AH, AHS, Alberta Office of Chief Medical Officer of Health, First Nations Inuit health Branch, CLS, Clinical Informatics – AH, ProvLab Alberta), universities (University of Calgary – Alberta Children’s Hospital Research Institute; University of Alberta – Women and Children’s Health Research Institute), and community stakeholders (Alberta Children’s Hospital Parent Advisory Committee). APPETITE’s Executive team will seek advice from its SAC on dispute resolution for high-level issues.

#### International Scientific Advisory Committee (ISAC)

This group includes experts in enteric bacteria (Dr. Claire Jenkins, Public Health England), diagnostic microbiology (Dr. Carey-Ann Burnham, Washington University), translational research (Dr. Eileen Klein, Seattle Children’s Hospital), emerging topics in gastrointestinal research (Dr. Phil Sherman – Director, Canadian Institutes of Health Research, Institute of Nutrition Metabolism and Diabetes), viral gastroenteritis (Dr. Jon Gentsch, Centers for Disease Control and Prevention), virology [Dr. Marion Koopmans, National Institute of Public health (Netherlands)] They are also joined by Dr. Bill Ghali who has expertise in leading large multi-disciplinary research teams within the University of Calgary (U of C). They will serve as strategic and scientific advisors to APPETITE’s research programs and collaborating members. APPETITE will host each ISAC member as visiting professors for periods of up to 1 week, during which time they will advise in real-time on APPETITE activities, and specific projects, give local teaching/training rounds and will meet with trainees.

#### APPETITE executive

Composed of APPETITE’s Lead (Dr. Freedman), Co-Leads (Drs. Lee, Louie, Pang), and the health economic activity Co-Lead (Dr. Currie), the executive committee meets weekly. Its members are accountable for the core decision-making of all programs, ensuring completeness of agency reporting, human resource and financial matters, keeping all activities on track, and updating team members and relevant end-users as required.

Future directions

Our comprehensive and extensive dataset will contain the necessary information to engage in deep, evidence-informed, discussions regarding policy change with our partner decision-makers at Alberta’s laboratories and the Office of the Chief Medical Officer of Health. These discussions will determine diagnostic testing, vaccine, and surveillance policy in Alberta for years to come, thereby optimizing health at individual and societal levels. APPETITE’s data and specimen repository will be employed to conduct the next steps in PCR-based, point of care diagnostics and norovirus vaccine research. Academia and industry partners will work to develop: 1) an oral viral pathogen panel; and 2) simplified point-of-care diagnostic technology [2, 3, 56] to enable use in remote communities and in low and middle income countries to reduce AGE mortality [57].

## Abbreviations

ACH: Alberta Children’s Hospital
AGE: Acute Gastroenteritis
AH: Alberta Health
AHS: Alberta Health Services
APPETITE: Alberta Provincial Pediatric EnTeric Infection TEam
CLS: Calgary Laboratory Services
ED: Emergency Department
GPP: Gastrointestinal Pathogen Panel
HCP: Health-Care Provider
IDSA: Infectious Disease Society of America
iKT: Integrated Knowledge Transfer
ISAC: International Scientific Advisory Committee
PCR: Polymerase Chain Reaction
PERC: Pediatric Emergency Research Canada
ProvLab: Provincial Laboratory for Public Health
SAC: Strategic Advisory Committee
TREKK: TRanslating Emergency Knowledge for Kids
